# Prognostic nutrition index (PNI) level in predicting surgical incision complications following medial opening-wedge high tibial osteotomy (MOWHTO) for knee osteoarthritis (KOA): A retrospective cohort study

**DOI:** 10.1097/MD.0000000000043770

**Published:** 2025-08-15

**Authors:** Jiaxiang Cheng, Rui Wang, Hang Su, Chenni Ji, Jiahao Zhang, Jingliang Zhang, Yanbin Zhu, Lei Zhang

**Affiliations:** a Department of Orthopedic Surgery, Cangzhou Central Hospital, Cangzhou, Hebei Province, People’s Republic of China; b Department of Orthopaedic Surgery, Huai’an Hospital of Huai’an City, Huai’an, Jiangsu Province, People’s Republic of China; c Department of Orthopaedic Surgery, Hebei Medical University Third Hospital, Shijiazhuang, Hebei Province, People’s Republic of China.

**Keywords:** knee osteoarthritis, malnutrition, medial opening-wedge high tibial osteotomy, prognostic nutrition index, surgical incision complications

## Abstract

Malnutrition is prevalent among hospitalized patients and has been shown to predict postoperative adverse events. This study aims to examine the relationship between malnutrition indicated by prognostic nutrition index (PNI) and surgical incision complication (SIC) following medial opening-wedge high tibial osteotomy (MOWHTO) for unicompartmental knee osteoarthritic (KOA). This was a retrospective study of patients who had undergone MOWHTO for varus KOA from January 2021 to June 2024 in 2 hospitals. Baseline data and laboratory test results were collected by reviewing the inpatient medical records. The outcome, incidence of SIC within 30 days postoperatively, was identified by reviewing the inpatient medical records and post-discharge outpatient follow-up records. The restricted cubic spline curve, receiver operating characteristic curve, univariate tests and multivariate logistic regression models were employed to identify the relationship between PNI and SIC. 528 patients were enrolled, comprising 190 males and 338 females, with an average age of 56.2 ± 6.5 years. Within the 30 days postoperatively, 48 (rate, 9.1%; 95% confidence interval (CI) = 6.6%–11.5%) patients developed SICs. The adjusted restricted cubic spline models demonstrated a significant inverted “J-shaped” nonlinear relationship (*P* < .05). The receiver operating characteristic curve analysis revealed a cutoff value for PNI of 47.0, accordingly categorizing patients into low PNI (n = 141) and high PNI (n = 387) groups. The multivariate models, using different adjustment methods, indicated a significant relationship between PNI < 47.0 and an increased risk of SICs, odds ratio of 4.86 (95% CI = 2.12 to 11.11) for the “enter” method and of 4.12 (95% CI = 2.23 to 7.61) for the “backward elimination” method. This study elucidates the significant relationship between PNI and SICs following MOWHTO for varus KOA. These findings emphasize the need for early nutritional assessment to improve patient safety and enhance surgical results.

## 1. Introduction

High tibial osteotomy is a well-established procedure for treating varus unicompartmental knee osteoarthritis (KOA).^[1,2]^ In the past decade, opening-wedge high tibial osteotomy (MOWHTO) has regained popularity due to its relatively lower technical demands, precise knee alignment, rapid functional recovery, favorable patient-reported clinical outcomes, and excellent long-term survivorship.^[1,3,4]^ Nonetheless, the high incidence of postoperative complications remains a significant concern, with reported overall rates of up to 29.7%,^[5–7]^ and wound complications ranging from 1.9% to 18.0%.^[8,9]^ Consequently, patients who experience wound complications are at a substantially increased risk of deep infection, delayed or nonunion of the osteotomy gap, prolonged functional recovery, and may require unplanned readmission or reoperation.^[9,10]^

The impact of nutritional status on surgical outcomes has gained attention over the decades. On the one hand, malnutrition is highly prevalent among patients with KOA. A population-based study in India indicated a malnutrition prevalence of 69.5% upon hospital admission for these patients.^[11]^ On the other hand, clinical practice guidelines recommend nutritional assessment and interventions prior to surgery in some geriatric orthopedic fields.^[12,13]^ The prognostic nutrition index (PNI) is a well-established and extensively used indicator of malnutrition. It is a straightforward and easily calculable tool that integrates serum albumin levels – reflecting long-term nutritional status and lymphocyte counts -indicating recent nutritional and immune status, thereby theoretically providing insights into a patient’s comprehensive view of nutritional and immune status. In research, lower PNI values have demonstrated strong predictive capability for various adverse outcomes, such as increased postoperative morbidity and mortality across a range of surgical procedures.^[14–17]^ Regarding KOA, PNI has demonstrated strong predictive power for postoperative adverse events following knee arthroplasties.^[18,19]^ We hypothesize that this predictive ability is also applicable to surgical incision complication (SIC) after MOWHTO for KOA. To date, we are unaware of any relevant reports on this topic.

This study aims to explore the relationship between PNI and SIC in patients undergoing MWOHTO for KOA by: visually illustrating the dose-effect relationship between PNI and SIC using multivariate restricted cubic curves; determining the optimal cutoff value of PNI that best predicts the overall risk of complications; and assessing whether the effect of PNI on SICs is independent.

## 2. Materials and methods

This was a retrospective observational study. The study protocol was approved by the ethics committee of the Cangzhou Central Hospital and Hebei Medical University Third Hospital before its commencement; the committee waived the requirement for informed consent due to the retrospective nature of the research and the anonymity of patient data. The study was conducted in accordance with the Helsinki Declaration.

Two researchers (JC. and CJ.) retrieved and reviewed the inpatient medical records of adult patients aged 40 years or older with a primary diagnosis of KOA and receiving a subsequent MOWHTO procedure in either hospital between January 2021 and June 2024. Inclusion criteria for the study were: meeting the diagnostic criteria for KOA and undergoing MOWHTO for the first time; the completion of plasma albumin levels and lymphocyte count testing within 2 days of admission prior to surgery; and complete medical records and postoperative 1-month follow-up data. Patients were excluded if they had undergone previous knee joint surgery or revision surgery; acute or chronic infection (systemic or local) prior to surgery; received exogenous albumin supplementation, or any component or whole blood transfusion after admission but before surgery; chronic use of glucocorticoids or immunodeficiency; concurrent or additional surgeries (except arthroscopic knee procedures) during index hospital stay; occurrence of other surgery-related complications during hospitalization or follow-up; or had incomplete medical records or 1-month follow-assessments.

### 2.1. Procedure and perioperative management

All surgeries began with a standard arthroscopic examination to assess cartilage and meniscal damage, and if necessary, a repair or partial resection procedure was performed for meniscal tears. The MOWHTO procedure was carried out using the biplanar osteotomy technique according to the standard methods.^[20]^ In brief, the preoperative plan involved shifting the mechanical axis to a point 57% lateral on the transverse diameter of the tibial plateau. The osteotomy site was opened to the planned correction width with intraoperative fluoroscopic limb alignment assessment. As deemed necessary, for cases with larger gaps or those considered at-risk of malunion by the treating surgeons, autologous iliac bone strips, allogeneic bone, synthetic bone, or other grafting materials were used to fill the osteotomy gap. The osteotomy site was stabilized using T-shaped plates with locking screws (Shandong Weigao Orthopedic Materials Co., Ltd.; Double Medical Technology Co., Ltd.). Prophylactic antibiotics (generally, 1 g of cefazolin) was administered 30 minutes prior to skin incision. For procedures lasting over 3 hours, an additional dose was provided. Postoperatively, antibiotic prophylaxis was not standardized and was primarily based on the individual patient’s risk of infection as assessed by the treating surgeons, alongside the management of drainage tube placement and removal timing.

A drainage tube was placed in all patients and removed on day 2 postoperatively. A prophylactic dose of enoxaparin was administered subcutaneously at a dose of 4000 IU once daily, starting from 12 hours postoperatively. Postoperatively, isometric quadriceps and active ankle exercises were initiated after surgery without immobilization; non-weight-bearing mobilization on crutches began at postoperative day 2 and continued for the first 4 weeks, followed by partial-weight-bearing mobilization during week 5 and week 6, and full-weight-bearing thereafter.

### 2.2. Identification of exposure and outcomes

Laboratory blood sampling and testing were performed according to the manufacturer’s instructions, on the first morning after admission. The PNI was calculated as follows: PNI = 10 × serum albumin (g/dl) + 5 × lymphocyte (10^9^/L).^[21]^ Typically, the normal value range for PNI is 50 to 65,^[22,23]^ while 40 is usually considered the cutoff point for malnutrition.^[23]^ SICs were defined as poor healing of superficial incisions accompanied by inflammatory reactions at the incision (mainly including dehiscence of the incision, fat liquefaction, exudation, hematoma, delayed healing, and necrosis of skin margins), as proposed by Dennis et al^[24]^ in the context of arthroplasty, and also included both superficial and deep surgical site infections. Notably, while bleeding and wound secretion are common occurrences in the immediate postoperative phase, hematomas that persist beyond the initial 72 hours post-surgery – often accompanied by significant swelling and pain – were classified as a SIC. Data on SICs were gathered by reviewing inpatient medical records and outpatient follow-up data from the 30 days post-discharge; if there were relevant descriptions, clear diagnoses, or documentation, patients were classified as having SICs.

### 2.3. Variables of interest

The data collected included: demographics (age, sex, body mass index, and residence), history of comorbidities or conditions (hypertension, diabetes mellitus, chronic obstructive pulmonary disease, cardiovascular disease, liver disease, renal insufficiency, hyperlipidemia, hypercholesteremia, any past operations, alcohol consumption, and smoking), surgery-related variables (American Society of Anesthesiologists classification, length of incision, opening width, sidedness, time to operation, anesthesia technique, surgical duration in minutes, intraoperative bleeding, bone graft), laboratory results at admission (above-mentioned serum albumin and lymphocyte count, as well as fasting blood glucose, red blood cell count, white blood cell count, platelet count, hemoglobin concentration, sodium concentration), and overall and postoperative length of hospital stay.

### 2.4. Statistical analysis

Continuous variables are presented as mean ± standard deviation, with normality assessed using the Kolmogorov–Smirnov test. For normally distributed variables, independent-sample Student *t* test is used; otherwise, the Mann–Whitney test is used. Categorical variables are presented as frequency and percentage, analyzed using the Chi-square test or Fisher’s exact test, as appropriate.

A restricted cubic spline (RCS) with 4 knots, adjusted for all listed covariates, was employed to visually examine the dose-effect relationship between the PNI and the risk of SIC. A *P* < .05 indicates that the significant nonlinear model provides a better fit for the data. The PNI value at which the odds ratio (OR) for SICs equals 1 was considered as a potential cutoff point. Additionally, the receiver operating characteristic (ROC) curve analysis was performed to determine the potential optimal cutoff value for predicting SIC, maximizing the Youden index (sensitivity + specificity − 1). For clinical purposes, the optimal cutoff value was established, allowing classification of patients into low- or high PNI groups. The incidence rate of SIC in respective group was calculated, and the post hoc statistical power was calculated using the G*power software (version 3.1.9.2, Universität Kiel, Kiel, Germany).

Patients with low- or high-PNI value were compared using the aforementioned univariate analyses. Variables with *P* < .10 that might significantly influence the PNI exposure were subsequently included in a multivariate logistic regression model. Factors such as demographics (age, sex), and other well-established variables known to affect the SICs, as reported in the literature were also included, regardless of their statistical significance related to PNI in this study. Two methods were used for multivariate model: the “enter” selection method (namely, the totally adjusted model), and the stepwise backward elimination method. The magnitude of association was indicated using OR and 95% confidence interval (CI). The goodness-of-fit of the multivariate model was evaluated using the Hosmer−Lemeshow (H−L) test, with *P* > .05 indicating an acceptable fit. A *P* < .05 was considered statistically significant. All statistical analyses were performed using SPSS 27.0 (IBM, Armonk, New York).

## 3. Results

A total of 528 eligible patients participated in the study, comprising 190 (36.0%) males and 338 (64.0%) females, with an average age of 56.2 ± 6.5 years. Within the 30 days postoperatively, 48 patients developed SICs, resulting in an overall rate of 9.1% (95% CI = 6.6%–11.5%). Most patients with complications healed uneventfully, aided by careful wound dressing changes. Six cases of superficial infection and 2 cases of deep surgical site infection were identified, which were treated with intravenous antibiotics; none required surgical debridement.

**Figure 1.**
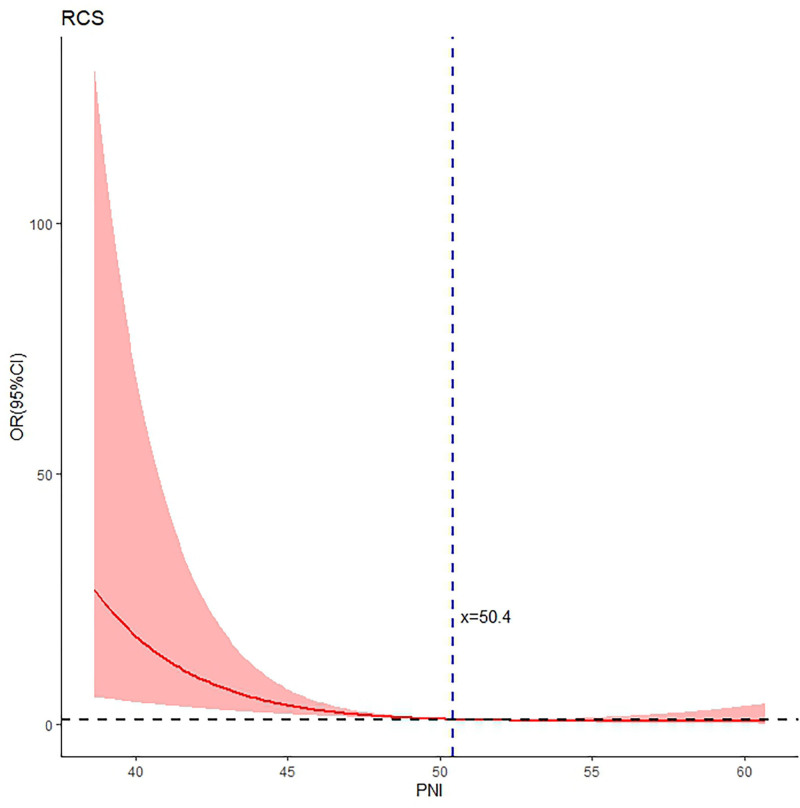
Unadjusted RCS curve depicting the relationship between PNI and the risk of incision complications following MOWHTO for varus KOA, indicting the significant nonlinear “inverted J-shaped” curve, with cutoff value of 50.4 which corresponds to OR = 1. CI = confidence interval, KOA = knee osteoarthritis, MOWHTO = medial opening-wedge high tibial osteotomy, OR = odds ratio, PNI = prognostic nutrition index, RCS = restricted cubic spline.

**Figure 2.**
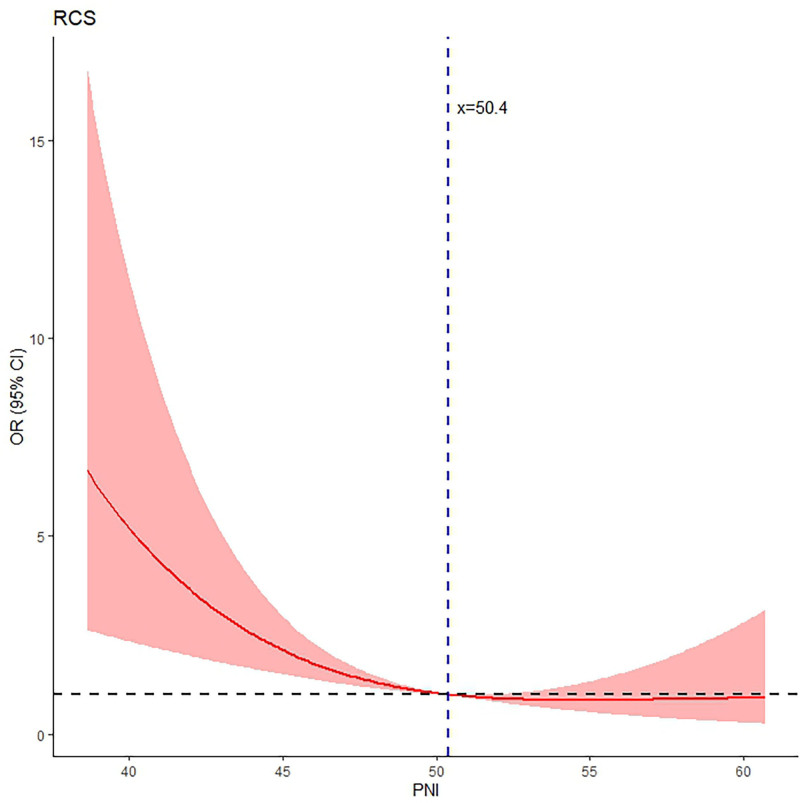
RCS curve adjusted for all variables listed in the text, showing the consistently significant nonlinear “inverted J-shaped” curve, with cutoff value of 50.4 which corresponds to OR = 1. CI = confidence interval, OR = odds ratio, PNI = prognostic nutrition index, RCS = restricted cubic spline.

The average albumin level was 41.6 ± 3.9 g/L, the lymphocyte count 1.8 ± 0.5 *10^9^/L, and the PNI 50.4 ± 5.4. As shown in Figures 1 and 2, both the unadjusted and adjusted RCS models demonstrated a remarkable decreasing trend in the risk of SIC as the PNI score increased, exhibiting an inverted “J-shaped” relationship. The reference point (OR = 1) was observed at a PNI of 50.4; and the test for nonlinearity was statistically significant (*P* < .05).

**Figure 3.**
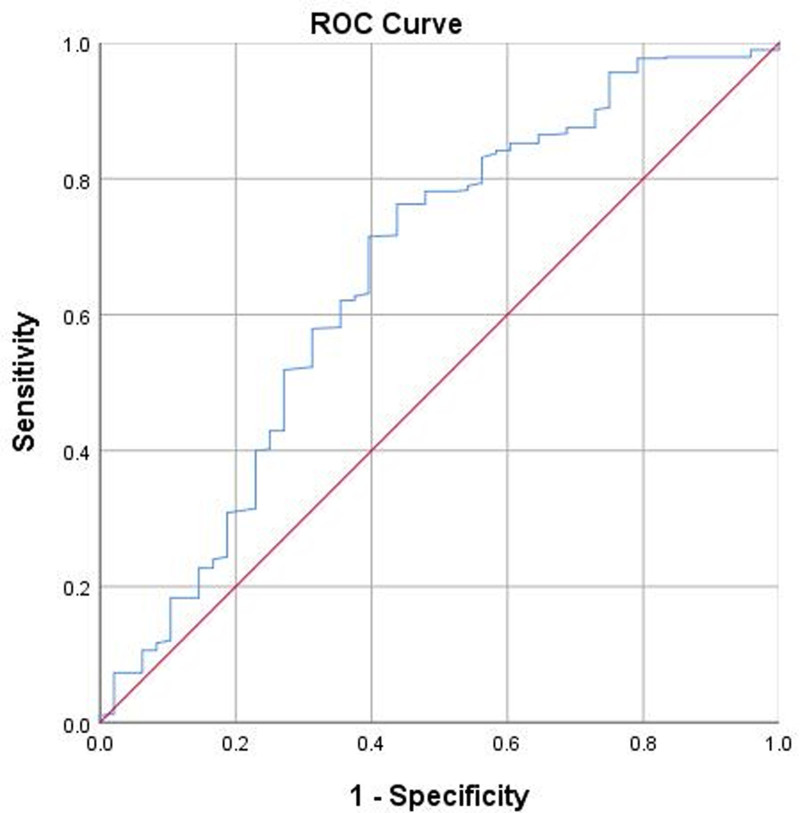
The ROC curve used to identify the optimal cutoff value of PNI for predicting incision complication risk to categorize patients. The optimal cutoff value was 47.0, corresponding to a sensitivity of 0.763 and a specificity of 0.562, and the area under the curve was 0.660 (*P* < .001). PNI = prognostic nutrition index, ROC = receiver operating characteristic.

The ROC curve analysis revealed a cutoff value for PNI of 47.0, which corresponded to a sensitivity of 0.763 and a specificity of 0.562. The area under the curve was 0.660 (*P* < .001; Fig. 3). For clinical application purposes, a PNI of 47.0 was established as the optimal cutoff point, categorizing patients into low PNI (<47.0, n = 141) and high PNI (≥47.0, n = 387) groups, with average PNI values of 43.6 ± 2.8 and 52.9 ± 3.7, respectively (*P* < .001 for the difference). The unadjusted OR for SIC in the low PNI group (incidence of complications, 19.1%, 27/141) compared to the high PNI group (incidence of complications, 5.4%, 21/387) was 4.13 (95% CI = 2.25 to 7.58). The post hoc statistical power calculation, based on a calculated large effect size of 0.475 (Cohen’s w), indicated a power higher than 99.9% to detect a clinically meaningful difference in the incidence rate of SIC.

As shown in Table 1, patients with low PNI had a trend towards a higher prevalence of chronic obstructive pulmonary disease (9.2% vs 4.9%, *P* = .066), and were less likely to have hypercholesteremia (1.4% vs 7.5%, *P* = .009), had fewer lengths of incision ≥8 mm (17.7% vs 26.4%, *P* = .040), reduced intraoperative bleeding (109.8 ± 31.8 vs 126.5 ± 62.5 ml), lower platelet counts (221.0 ± 67.7 vs 231.6 ± 52.9 *10^12^/L, *P* = .042), RBC count (3.9 ± 0.5 vs 4.5 ± 0.4 *10^12^/L, *P* < .001), and hemoglobin level (116.9 ± 15.8 vs 133.5.3 ± 12.3, *P* < .001).

**Table 1:** Comparison of demographic and clinical characteristics between high- and low-PNI groups.

Variables	PNI ≥ 47 (n = 387)	PNI < 47 (n = 141)	*P*
Age (yr)	56.3 ± 6.5	56.0 ± 6.4	.641
<60	266 (68.7)	99 (70.2)	.745
≥60	121 (31.3)	42 (29.8)	
Sex (male)	140 (36.2)	50 (35.5)	.880
Residence (rural)	204 (52.7)	77 (54.6)	.699
BMI (kg/m^2^)	26.9 ± 3.5	26.4 ± 3.2	.143
<24.0	79 (20.4)	34 (24.1)	.389
24.0–27.9	181 (46.8)	71 (50.4)	
28.0–31.9	101 (26.1)	27 (19.1)	
≥32.0	26 (6.7)	9 (6.4)	
Hypertension	142 (36.7)	54 (38.3)	.736
Diabetes mellitus	39 (10.1)	12 (8.5)	.590
Cardiovascular disease	51 (13.2)	14 (9.9)	.315
COPD	19 (4.9)	13 (9.2)	.066
Liver disease	44 (11.4)	15 (10.6)	.813
Smoking			.784
Current	46 (11.9)	18 (12.8)	
Former or never	341 (88.1)	123 (87.2)	
Alcohol consumption			.404
Current	47 (12.1)	21 (14.9)	
Former or never			
Renal insufficiency	29 (7.5)	13 (9.2)	.517
Hyperlipidemia	65 (16.8)	21 (14.9)	.600
Hypercholesterolemia	29 (7.5)	2 (1.4)	.009
History of any operation (except for knee)	109 (28.2)	35 (24.8)	.445
ASA classification			.016
I or II	340 (87.9)	134 (95.0)	
III or more	47 (12.1)	7 (5.0)	
Opening width (≥10 mm)	284 (73.4)	100 (70.9)	.574
Length of incision (≥8 mm)	102 (26.4)	25 (17.7)	.040
Sidedness			.521
Left	207 (53.5)	72 (51.1)	
Right	180 (46.5)	69 (48.9)	
Time from admission to operation (d)			
Anesthesia technique (general)	13 (3.4)	6 (4.3)	.625
Surgical duration (min)			.144
<90	109 (28.2)	49 (34.8)	
≥90	278 (71.8)	92 (65.2)	
Intraoperative bleeding (mL)	126.5 ± 62.8	109.8 ± 31.8	.003
Bone graft			.165
None or autograft	8 (2.1)	7 (5.0)	
Allograft	242 (62.5)	90 (63.8)	
Synthetic bone graft	42 (10.9)	18 (12.8)	
Others	95 (24.5)	26 (18.4)	
FBG (mmol/L)	5.6 ± 1.4	5.5 ± 1.0	.326
Platelet count (*10^12^/L)	231.6 ± 52.9	221.0 ± 67.7	.042
RBC count (*10^12^/L)	4.5 ± 0.4	3.9 ± 0.5	<.001
Hemoglobin (g/L)	133.5.3 ± 12.3	116.9 ± 15.8	<.001
Sodium concentration (mmol/L)	140.5 ± 2.1	140.7 ± 2.1	.333
WBC count (*10^9^/L)	6.1 ± 1.6	6.1 ± 1.8	.928
Overall length of hospital stay (d)	7.7 ± 2.9	7.4 ± 2.6	.282
Postoperative length of hospital stay (d)	5.1 ± 2.1	4.7 ± 1.8	.065

ASA = American Society of Anesthesiologists, BMI = body mass index, COPD = chronic obstructive pulmonary disease, FBG = fasting blood glucose, PNI = prognostic nutritional index, RBC = red blood cell, WBC = white blood cell.

Both the fully adjusted multivariate model and backward selection model indicated a significant relationship between PNI < 47.0 and an increased risk of SIC, with ORs of 4.86 (95% CI = 2.12 to 11.11) (Table 2) and of 4.12 (95% CI = 2.23 to 7.61) (Table 3), respectively. The both H–L tests showed an acceptable fit for both models (both *P* > .05).

**Table 2:** Multivariate analysis of PNI in association with postoperative incision complications after MWOHTO for KOA using “totally adjusted” logistic regression model.

Variables	β	Standard error	OR and 95% CI	*P*
PNI (<47.0 vs ≥47)	1.582	0.421	4.86 (2.13–11.11)	<.001
Age (yr)	−0.040	0.026	0.96 (0.91–1.01)	.121
Sex (female vs male)	0.296	0.438	1.34 (0.57–3.17)	.499
COPD	−1.519	1.056	0.22 (0.03–1.74)	.151
Renal disease	1.273	0.447	3.57 (1.49–8.57)	.004
Hypercholesterolemia	0.012	0.795	1.01 (0.21–4.8)	.988
ASA group (3 or 4 vs 1or 2 or)	−1.357	1.048	0.26 (0.03–2.01)	.196
Smoking (current vs former or never)	−0.231	0.547	0.79 (0.27–2.32)	.673
Blood loss	0.000	0.003	1.00 (0.99–1.01)	.964
Surgical duration (≥90 vs <90 min)	−0.171	0.171	0.84 (0.60–1.18)	.318
Length of incision (≥8 mm)	0.204	0.380	1.23 (0.58–2.58)	.592
RBC count (*10^12^/L)	−0.334	0.543	0.72 (0.25–2.07)	.538
Hemoglobin (g/L)	0.019	0.018	1.02 (0.98–1.06)	.291

β, regression coefficient.

ASA = American Society of Anesthesiologists, CI = confidence interval, COPD = chronic obstructive pulmonary disease, KOA = knee osteoarthritis, MWOHTO = medial opening-wedge high tibial osteotomy, OR = odds ratio, PNI = prognostic nutritional index, RBC = red blood cell.

**Table 3:** Logistic regression analysis for PNI associated with postoperative incision complications after MWOHTO for KOA using stepwise backward method.

Variables	β	Standard error	*P*	Variables
PNI (<47.0 vs ≥47)	1.416	0.313	4.12 (2.23–7.61)	<0.001
Renal disease	1.135	0.428	3.11 (1.34–7.21)	0.008

β, regression coefficient.

KOA = knee osteoarthritis, MWOHTO = medial opening-wedge high tibial osteotomy, PNI = prognostic nutritional index.

## 4. Discussion

The main findings included an inverted “J-shaped” nonlinear curve after multivariate adjustment, the identification of cutoff points for PNI at 47.0 and 50.4, and an OR of 4.12 for risk of SIC in patients with low PNI compared to those with PNI ≥ 47.0.

The prominent feature observed in both the univariate and multivariate RCS analyses is the inverted “J-shaped” nonlinear curve. This quantifiable curve is helpful in distinctively understanding the relationship between PNI and the risk of SIC, and the consistency observed between the univariate and multivariate RCS analyses strengthens the robustness of the findings and underscores the independent effect of PNI on the risk of SIC. The PNI threshold of 50.4 derived from RCS represents a critical point that delineates the protective and high-risk zones for SIC. Interestingly, 50.4 is higher than the alternative classification threshold of 47.0 that was determined through the ROC curve analysis, which is predictable due to the inherent characteristics of these analytic approaches. The difference of 3.4 between them may serve a “buffer zone,” alerting clinicians to the increasing risk of SIC and also providing an opportunity to closely monitor patients’ long-term nutritional status, recent infection status or immune function and to proactively manage any underlying conditions.

The relationship between malnutrition and adverse events following surgeries has been well studied in a range of orthopedic subspecialties. Huang et al^[25]^ prospectively examined 2161 patients undergoing total joint arthroplasty with either an abnormal serum albumin or transferrin for indicating malnutrition, and found a 12% overall rate of complication in the malnourished group, as compared to 2.9% in group with normal parameters. Additionally, they found malnutrition could predict serious complications such as infection, renal, and cardiac complications. Furthermore, in elderly patients undergoing elective or urgent orthopedic surgeries (e.g., hip fracture repair or arthroplasty), both the prevalence of malnutrition and its impact on adverse events are more pronounced,^[26,27]^ primarily due to their age-related physiological declines and the increased vulnerability to malnutrition. Our finding that a low PNI is associated with a 4.13-fold increase in the risk of SIC supports the existing literature that underscores the critical role of nutritional status in surgical outcomes. However, it is important to note that the predictive value of preoperative PNI is clearly greater than that of postoperative PNI. This is due to the significant dynamic changes in postoperative albumin levels and lymphocyte counts – particularly the latter – which are influenced by factors such as the stress response to surgical trauma, fluctuations in blood volume due to blood loss and transfusions, and implantation of foreign materials, such as internal fixation devices or grafts.^[28,29]^

Understanding the underlying mechanisms that link PNI to SIC is not difficult, and several explanations can be proposed. First, albumin serves as a key indicator of chronic nutritional status due to its long half-life (15–19 days). Preoperative hypoalbuminemia suggests chronic malnutrition, which can impair fibroblast proliferation and subsequently reduce collagen synthesis, hindering the healing of incisional wounds.^[30]^ Second, a decrease in plasma albumin can lead to a reduction in plasma colloid osmotic pressure, resulting in edema in the surrounding tissues and thus increasing the risk of incision complications.^[31]^ Third, hypoalbuminemia is associated with immunosuppression and a lower lymphocyte count, reflecting a recent inflammatory state or a decline in the body’s immune response.^[32]^ Collectively, these factors contribute to the heightened risk of incision complications in patients with low PNI. Accordingly, PNI may serve as a more suitable index than either of the 2 individual measures in predicting SICs, and the strong association indicated by an OR of 4.12 support this assertion.

These findings have important clinical implications, underscoring the essential role of early nutritional assessment in patients with osteoarthritis prior to undergoing the MOWHTO procedure, particularly for vulnerable individuals who are older and have underlying comorbidities. By recognizing the critical PNI thresholds of 47.0 and 50.4, clinicians can better assess an individual patient’s risk of SICs, thereby facilitating shared decision-making and enabling proactive interventions or preventive measures. Furthermore, integrating PNI into preoperative evaluations, in conjunction with other predictive indicators, allows clinicians to address the specific needs of at-risk patients.

This study suffered from several limitations. First, the retrospective nature of this study introduces inherent limitation in data collection, affecting data precision and the potential for selection bias. Second, the strict inclusion and exclusion criteria may have led to an overestimation of the incidence of SICs, as patients with concurrent medical conditions or other surgical-related complications were excluded. However, as a trade-off, this strategy allowed us to refine the relationship between PNI and SIC. Third, several variables directly related to wound care – such as hair removal techniques, medication administration, skin closure, and postoperative knee exercises – were not captured, which may have influenced the observed association. Additionally, other unconsidered factors could also have impacted the results, leaving residual confounding present. Fourth, given that the study was conducted at a university-affiliated hospital and a regional comprehensive tertiary referral center, the findings may not accurately represent the average standard of care for treating such conditions, and thus our findings may be less generalizable to other settings or populations.

In summary, this study elucidates the significant relationship between the preoperative PNI and the postoperative SICs in patients undergoing MOWHTO for varus KOA. These findings emphasize the need for early nutritional assessment in this population and advocate for the integration of PNI into preoperative evaluation, to improve patient safety and enhance surgical results.

## Acknowledgments

We are grateful to C.L. and S.L. of Department of Orthopaedics Surgery for their kind help.

## Author contributions

**Conceptualization:** Lei Zhang.

**Data curation:** Jiaxiang Cheng, Jingliang Zhang.

**Formal analysis:** Jiaxiang Cheng, Rui Wang, Hang Su, Jingliang Zhang.

**Funding acquisition:** Chenni Ji.

**Investigation:** Hang Su, Jiahao Zhang, Jingliang Zhang, Yanbin Zhu.

**Methodology:** Jiaxiang Cheng, Hang Su, Chenni Ji, Jiahao Zhang, Yanbin Zhu.

**Resources:** Chenni Ji, Jiahao Zhang, Lei Zhang.

**Software:** Jiaxiang Cheng, Rui Wang, Chenni Ji, Jiahao Zhang.

**Supervision:** Chenni Ji, Yanbin Zhu, Lei Zhang.

**Validation:** Jiaxiang Cheng, Jiahao Zhang, Yanbin Zhu.

**Writing – original draft:** Jiaxiang Cheng.

**Writing – review & editing:** Yanbin Zhu, Lei Zhang.

## References

[1] Dal Fabbro G Grassi A Agostinone P. High survivorship rate and good clinical outcomes after high tibial osteotomy in patients with radiological advanced medial knee osteoarthritis: a systematic review. Arch Orthop Trauma Surg. 2024;144:3977–88. 38430233 10.1007/s00402-024-05254-0 PMC11564305

[2] Gkekas NK Komnos GA Mylonas T Chalatsis G Koutalos AA Hantes ME. Medial open wedge high tibial osteotomy is a viable option in young patients with advanced arthritis in a long-term follow-up. Knee Surg Sports Traumatol Arthrosc. 2024;33:1025–32. 39290201 10.1002/ksa.12469 PMC11848967

[3] Wu L Lin J Jin Z Cai X Gao W. Comparison of clinical and radiological outcomes between opening-wedge and closing-wedge high tibial osteotomy: a comprehensive meta-analysis. PLoS One. 2017;12:e0171700. 28182736 10.1371/journal.pone.0171700 PMC5300239

[4] Wang Z Zeng Y She W Luo X Cai L. Is opening-wedge high tibial osteotomy superior to closing-wedge high tibial osteotomy in treatment of unicompartmental osteoarthritis? A meta-analysis of randomized controlled trials. Int J Surg. 2018;60:153–63. 30445197 10.1016/j.ijsu.2018.10.045

[5] Han SB In Y Oh KJ Song KY Yun ST Jang KM. Complications associated with medial opening-wedge high tibial osteotomy using a locking plate: a multicenter study. J Arthroplasty. 2019;34:439–45. 30503322 10.1016/j.arth.2018.11.009

[6] Seo SS Kim OG Seo JH Kim DH Kim YG Lee IS. Complications and short-term outcomes of medial opening wedge high tibial osteotomy using a locking plate for medial osteoarthritis of the knee. Knee Surg Relat Res. 2016;28:289–96. 27894176 10.5792/ksrr.16.028 PMC5134783

[7] Ollivier M Claes S Mabrouk A. Surgical strategy and complication management of osteotomy around the painful degenerative varus knee: ESSKA formal consensus Part II. Knee Surg Sports Traumatol Arthrosc. 2024;32:2194–205. 38769785 10.1002/ksa.12273

[8] Guo H Song B Zhou R. Risk factors and dynamic nomogram development for surgical site infection following open wedge high tibial osteotomy for varus knee osteoarthritis: a retrospective cohort study. Clin Interv Aging. 2023;18:2141–53. 38143487 10.2147/CIA.S436816 PMC10748744

[9] Liu T-W Chiu C-H Chen AC-Y Chang S-S Chan Y-S. Risk factor analysis for infection after medial open wedge high tibial osteotomy. J Clin Med. 2021;10:1727–35. 33923605 10.3390/jcm10081727 PMC8073483

[10] Seki K Sakka A Tokushige A Imagama T Mutou M Taguchi T. Treatment for Staphylococcus aureus infection following open wedge high tibial osteotomy using antibiotic-impregnated calcium phosphate cement. Knee Surg Sports Traumatol Arthrosc. 2014;22:2614–7. 23462956 10.1007/s00167-013-2460-9

[11] Maheshwari V Choudhury AK Yadav R Dhingra M Kant R Kalia RB. Prevalence of poor nutrition in knee osteoarthritis patients: a hospital-based cohort study in indian population. Indian J Orthop. 2024;58:298–307. 38425822 10.1007/s43465-023-01090-3 PMC10899134

[12] Briguglio M Wainwright TW. Nutritional and physical prehabilitation in elective orthopedic surgery: rationale and proposal for implementation. Ther Clin Risk Manag. 2022;18:21–30. 35023922 10.2147/TCRM.S341953 PMC8747789

[13] Rosenberger C Rechsteiner M Dietsche R Breidert M. Energy and protein intake in 330 geriatric orthopaedic patients: are the current nutrition guidelines applicable? Clin Nutr ESPEN. 2019;29:86–91. 30661706 10.1016/j.clnesp.2018.11.016

[14] Bullock AF Greenley SL McKenzie GAG Paton LW Johnson MJ. Relationship between markers of malnutrition and clinical outcomes in older adults with cancer: systematic review, narrative synthesis and meta-analysis. Eur J Clin Nutr. 2020;74:1519–35. 32366995 10.1038/s41430-020-0629-0 PMC7606134

[15] Seo YJ Yu J Park JY. Prognostic nutritional index and postoperative pulmonary complications in patients with major burns. J Surg Res. 2022;279:453–63. 35841814 10.1016/j.jss.2022.06.038

[16] Wang Y Jiang Y Luo Y. Prognostic nutritional index with postoperative complications and 2-year mortality in hip fracture patients: an observational cohort study. Int J Surg. 2023;109:3395–406. 37526114 10.1097/JS9.0000000000000614 PMC10651254

[17] Tunçez M Bulut T Süner U Önder Y Kazimoğlu C. Prognostic nutritional index (PNI) is an independent risk factor for the postoperative mortality in geriatric patients undergoing hip arthroplasty for femoral neck fracture? A prospective controlled study. Arch Orthop Trauma Surg. 2024;144:1289–95. 38265465 10.1007/s00402-024-05201-z

[18] Hanada M Hotta K Matsuyama Y. Prognostic nutritional index as a risk factor for aseptic wound complications after total knee arthroplasty. J Orthop Sci. 2021;26:827–30. 32883543 10.1016/j.jos.2020.07.019

[19] Hotta K Hanada M Matsuyama Y. Impact of prognostic nutritional index on the occurrence of post-operative delirium after total knee arthroplasty. J Jt Surg Res. 2024;2:71–6.

[20] Lobenhoffer P Agneskirchner JD. Improvements in surgical technique of valgus high tibial osteotomy. Knee Surg Sports Traumatol Arthrosc. 2003;11:132–8. 12774149 10.1007/s00167-002-0334-7

[21] Hong J Huang QQ Liu WY. Three nutritional indices are effective predictors of mortality in patients with type 2 diabetes and foot ulcers. Front Nutr. 2022;9:851274. 35369056 10.3389/fnut.2022.851274 PMC8965352

[22] Yang G Wang D He L. Normal reference intervals of prognostic nutritional index in healthy adults: a large multi-center observational study from Western China. J Clin Lab Anal. 2021;35:e23830. 34018637 10.1002/jcla.23830 PMC8274996

[23] Onodera T Goseki N Kosaki G. Prognostic nutritional index in gastrointestinal surgery of malnourished cancer patients. Nihon Geka Gakkai Zasshi. 1984;85:1001–5. 6438478

[24] Dennis DA. Wound complications in total knee arthroplasty. Orthopedics. 1997;20:837–40. 9306466 10.3928/0147-7447-19970901-26

[25] Huang R Greenky M Kerr GJ Austin MS Parvizi J. The effect of malnutrition on patients undergoing elective joint arthroplasty. J Arthroplasty. 2013;28(8 Suppl):21–4. 23993346 10.1016/j.arth.2013.05.038

[26] Fernández Miró M Cabrejo Gavidia V Carrascosa Piquer O Valero Lanau J Toapanta Valencia M Aguado Jodar A. Malnutrition is associated with postoperative complications in elderly patients undergoing total hip arthroplasty. Endocrinol Diabetes Nutr (Engl Ed). 2023;70(Suppl 3):59–66. 37640474 10.1016/j.endien.2023.06.003

[27] Bohl DD Shen MR Hannon CP Fillingham YA Darrith B Della Valle CJ. Serum albumin predicts survival and postoperative course following surgery for geriatric hip fracture. J Bone Joint Surg Am. 2017;99:2110–8. 29257017 10.2106/JBJS.16.01620

[28] Sun J Yang G Yang C. Influence of postoperative hypoalbuminemia and human serum albumin supplementation on incision healing following total knee arthroplasty for knee osteoarthritis: a retrospective study. Sci Rep. 2024;14:17354. 39075140 10.1038/s41598-024-68482-9 PMC11286832

[29] Mahkovic-Hergouth K Kompan L. Is replacement of albumin in major abdominal surgery useful? J Clin Anesth. 2011;23:42–6. 21296246 10.1016/j.jclinane.2010.06.007

[30] Palmieri B Vadalà M Laurino C. Nutrition in wound healing: investigation of the molecular mechanisms, a narrative review. J Wound Care. 2019;28:683–93. 31600106 10.12968/jowc.2019.28.10.683

[31] Gonzales GB Njunge JM Gichuki BM. The role of albumin and the extracellular matrix on the pathophysiology of oedema formation in severe malnutrition. EBioMedicine. 2022;79:991–1005. 10.1016/j.ebiom.2022.103991 PMC9014367 35398787

[32] Manzoli TF Delgado AF Troster EJ. Lymphocyte count as a sign of immunoparalysis and its correlation with nutritional status in pediatric intensive care patients with sepsis: a pilot study. Clinics (Sao Paulo). 2016;71:644–9. 27982165 10.6061/clinics/2016(11)05 PMC5108166

